# Ecotoxicological Studies on the Action of Actara 25 WG Insecticide on Prussian Carp (*Carassius gibelio*) and Marsh Frog (*Pelophylax ridibundus*)

**DOI:** 10.3390/toxics10030114

**Published:** 2022-02-27

**Authors:** Alina Paunescu, Liliana Cristina Soare, Irina Fierascu, Radu Claudiu Fierascu, Cristina Florina Mihaescu, Lucica Tofan, Cristina Maria Ponepal

**Affiliations:** 1 Natural Science Department, Faculty of Sciences, Physical Education and Informatics, University of Pitesti, 110040 Pitesti, Romania; alina.paunescu@upit.ro (A.P.); cristina.soare@upit.ro (L.C.S.); cristina.mihaescu@upit.ro (C.F.M.); cristina.ponepal@upit.ro (C.M.P.); 2 National Research and Development Institute for Chemistry and Petrochemistry—ICECHIM, 060021 Bucharest, Romania; dumitriu.irina@yahoo.com (I.F.); radu_claudiu_fierascu@yahoo.com (R.C.F.); 3 Faculty of Natural and Agricultural Sciences, Ovidius University of Constanta, 900470 Constanta, Romania

**Keywords:** thiamethoxam, marsh frog, Prussian carp, oxygen consumption, respiratory rate, lung, pneumocytes

## Abstract

The toxic action of the Actara 25 WG insecticide (it contains 25% thiamethoxam as an active substance) in non-lethal doses was studied in two species of aquatic organisms—the Prussian carp (*Carassius gibelio*) and the marsh frog (*Pelophylax ridibundus*)—at two thermal levels, 6–8 °C (low temperature) and 18–20 °C (room temperature), respectively. In the Prussian carp, we recorded decreases in oxygen consumption and stimulation of the respiratory rhythm, changes that were more pronounced in the case of intoxicated fish and when the species were kept at room temperature. The histopathology of the lung in the frog illustrated the thickening of the conjunctival septum, an increase in the number of mucous cells, and an increase in the ratio between the diameter of the nucleus and the diameter of the pneumocyte. All of these changes were more pronounced in the animals kept at higher temperature. Our study looks at the extent to which temperature changes can influence the ability of poikilothermic organisms to withstand the presence of toxic substances in the environment as a result of the impact of the use of insecticides in agriculture. The two tested organisms are a common presence for the study area, which was affected in the last decade by climate change.

## 1. Introduction

Given the excessive growth of pest populations in agriculture, the use of insecticides is a phytosanitary indication that ensures increases in productivity. In this context, knowledge of the toxicity of insecticides is particularly important for human health, on the one hand, because they can bioaccumulate in fruits and vegetables and, on the other hand, because they reach the ground, are transported by rain, and are discharged into streams or rivers. Upon reaching the aquatic environment, they can cause harm to aquatic plant and animal species. Numerous researchers have pointed out their negative influence on the functioning of ecosystems. This is partly associated with the non-target effects observed in populations of pollinators (bees and bumblebees) and insectivorous birds [1]. With respect to the colony collapse disorder in 2013, the European Food Safety Authority restricted the use of the three most hazardous neonicotinoid insecticides: imidacloprid, thiamethoxam, and clothianidin [2]. Thiamethoxam has been partially banned by the European Commission due to its toxicity to honeybees [3].

The toxicity of an insecticide varies greatly depending on the species tested, with species having different degrees of sensitivity to different toxic substances; partition distribution is a critical factor affecting the toxicity of insecticides in animals [4]. Field levels of thiamethoxam can range from 0 to 15 ppb [5]. Detection of contamination of groundwater with thiamethoxam is only a matter of time; the levels measured for thiamethoxam in 2008 and 2009 in Wisconsin, for several wells, had values above 1 μg/L, with a maximum of 9 μg/L [6]. Thiamethoxam (average, 1.59; range, 0.20–8.93 μg/L) was detected at 23 monitoring locations over a five-year period; maximum concentrations detected in the water were 1490 ng/L [7]. Stability of thiamethoxam decreases with an increase of pH; **T_1/2_** (half-life) at pH 5–7 is >1 year, while it is only a few days at pH 9 [8].

Neonicotinoids are insecticides that are widely used in agriculture against sucking insects; however, they can also affect useful non-target insects such as honeybees [9]. Neonicotinoid insecticides have been in use since the 1990s [10]. They act on postsynaptic nicotinic acetylcholine receptors (nAChRs) [11].

Neonicotinoid insecticides also produce hepatotoxicity in mice [12,13], nephrotoxicity in mice [13], respiratory toxicity in mice [14], hyperglycemia in male rats [15], genotoxicity in rabbits [16], and endocrine disruption in birds [17]. Stara et al. [18] also showed harmful effects of neonicotinoid insecticides on mussels (*Mytilus galloprovincialis*). In another study, Stara [19] demonstrated that thiacloprid affected hemolymph biochemical parameters, cell viability in the digestive gland, and antioxidant biomarkers and lipid peroxidation in the digestive gland and gills on mussels at environmentally relevant concentrations (4.5 μg L^−1^).

The increasing use of neonicotinoid insecticides has led to the accumulation of their residues in surface waters; Morrissey cites 29 studies conducted in nine countries that reported detectable concentrations of this class of insecticides in puddles, streams, rivers, wetlands, and irrigation channels [20]. Another problem with neonicotinoid insecticides is that they persist in the environment for a very long time, over 20 years [21], so even if they are banned, their toxicity manifests itself over time.

Thiamethoxam is part of the family of neonicotinoid insecticides and is widely used both in agriculture and in horticulture or in pets to combat a wide range of insects. The insecticide thiamethoxam was first approved for use in agriculture by the US Environmental Protection Agency (USEPA) in 1999 [22]. These insecticides continue to appear in the market due to insufficient data on their toxicity to humans [23].

In the literature, toxicological tests with thiamethoxam for more than 30 freshwater species (insects, mollusks, crustaceans, algae, macrophytes, and fish) and four marine species (an alga, mollusk, crustacean, and fish) are reported [22]. There have been reports of various behavioral, hematological, and biochemical changes, along with reduced productivity, due to stress in animals even for short-term exposures to thiamethoxam [24,25].

In the organism, thiamethoxam is converted to clothianidin, which is more active than the parent molecules [26]. It acts as an antagonist of the postsynaptic receptor for acetylcholine, which is a neurotransmitter of the central nervous system, the parasympathetic nervous system, and some of the sympathetic system. The high insect toxicity of the pesticide is explained by the predominance of nicotinic receptors in the central nervous systems of these species [23].

Actara 25 WG is an insecticide containing 25% thiamethoxam as an active substance, accepted for use both on the ground and on leaf surfaces or seeds to combat a wide range of pests, including cuttlefish, aphids, white butterflies, and some species of cockroaches, including the Colorado cockroach [27]. Actara is a novel neonicotinoid insecticide belonging to a subclass of these nicotinyl compounds, and it is a systemic insecticide for soil and foliar applications [28]. Thiamethoxam (CGA 293433) belongs to the class of neonicotinoid insecticides, from the subclass of thianicotinyl substances.

In the present study, we studied the action of the Actara 25 WG insecticide (active substance: thiamethoxam, 25%) on the consumption of oxygen and respiratory rhythm in Prussian carp, as well as the lung injury induced by it in the marsh frog.

Fish have a reduced sensitivity to thiamethoxam, with observed acute median lethal and effect concentrations (LC_50_/EC_50_) ≥ 80 mg/L in all cases, which far exceeds surface water exposure concentrations [22]. Barbee and Stout, quoted from Fulton [29], reported a value of 114 mg/L thiamethoxam (LC50/96 h exposure) for bluegill sunfish and the value of 100 mg/L thiamethoxam for rainbow trout.

## 2. Materials and Methods

The biological material used were Prussian carp samples (*Carassius gibelio*), males and females, and marsh frogs (*Pelophylax ridibundus),* captured from the surrounding lakes of Piteşti.

After 10 days of adaptation in the lab, where they were fed ad libitum once a day, the fish were separated in lots, which were used separately for the following experiments:

### 2.1. The First Experiment Was Carried out with Prussian carp Individuals Separated into Four Lots, Each Lot Being Subdivided in Two Sublots (Ten Fish)

(1) Individuals younger than one year, with an average weight of 12.91 ± 1.74 g (C_0_);

(2) Individuals over one year old, with an average weight of 38.08 ± 4.57 g (C_1_).

The concentrations of Actara 25 WG used in the first experiment were 0.064, 0.128, 0.256, and 0.512 mg/L water, respectively (corresponding to thiamethoxam concentrations of 0.016, 0.032, 0.064, and 0.128 mg/L water).

For Prussian carp younger than one year (C_0_), only the lowest concentration of Actara 25 WG, 0.064 mg/L, was tested, respectively.

For individuals over one year were tested all the variants of insecticide concentrations.

The water temperature was between 18 and 20 °C.

### 2.2. The Second Experiment Was Carried out with Prussian carp Separated into Four Lots, Each Lot Being Subdivided in Two Sublots (Ten Fish)

(1) Individuals younger than one year, with an average weight of 11.33± 2.32 g (C_0_);

(2) Individuals over one year old with an average weight of 37.7± 4.56 g (C_1_).

The concentrations of Actara 25 WG used in the second experiment were 0.064, 0.128, 0.256, and 0.512 mg/L water, respectively, for the individuals over one year. For Prussian carp younger than one year (C_0_), only the lowest concentration of insecticide was tested.

The water temperature was between 6 and 8 °C. The concentrations that were used were established by study of the literature on thiamethoxam levels reached in surface waters and a preliminary survival test. The immersion of fish in these solutions was conducted after they had been well stirred and aired for five minutes. Fish were kept in 100 L glass aquaria with gently aerated tap water, at dissolved oxygen 7.50 ± 0.28 mg/L, pH 7.95 ± 0.75, total hardness 100 mg/L CaCO_3_ with a natural light: dark photoperiod.

In all experiments, the immersion solution was changed every 48 h by transferring the fish to another aquarium, and the water was continuously aired. The fish were fed during the experiments in order to avoid the intervention of this factor [30].

The pesticide concentration was verified by high-performance liquid chromatography (HPLC), using a Varian Pro Star chromatograph, (Column: Agilent Poroshell 120 EC C18, mobile phase sol A: 0.1 formic acid solution and B: acetonitrile). Measurements were performed with a Varian Liberty 110 spectrometer, using a five-point calibration curve [31]. For the administration of the toxic substance, stock solutions were prepared and analytically checked.

The energetic metabolism, expressed by the oxygen consumption, was determined using the closed respiratory chamber method (the oxygen dose in the water was established using the Winkler chemical method) by chemical dosing of dissolved oxygen in water with thiosulfate [30]. These determinations were made at intervals of 24, 48, 72, 96, 168, and 336 h, respectively. The breathing frequency was determined at the same intervals as in the case of the energetic metabolism.

Two weeks after exposure to Actara 25 WG solution with a concentration of 0.064 mg/L water, blood samples were taken from the caudal artery [30], determining the average number of erythrocytes (with Thoma chamber to Olympus microscope, according to the method described by Picoș and Năstăsescu [30]).

All the determinations were carried out in triplicates. Values are given as means ± standard error of the mean (S.E.M.). The data were analyzed for statistical significance using the analysis of variance (two-way ANOVA), followed by different post-hoc tests. Dunnett’s multiple comparison test was used in order to compare the means for each of the investigated parameters against their corresponding control group mean. The impact of the temperature on the toxic action was evaluated by pairwise comparisons between the mean values obtained for the groups receiving the same treatment at different temperatures, using Bonerroni’s method. The statistical analyses were performed using GraphPad Prism 7 (GraphPad Software Inc., La Jolla, CA, USA) software.

Correlation between oxygen consumption and respiration rate per minute was determined by calculating the Pearson correlation coefficient r (using the program SPSS for Windows) for the significance threshold *p* < 0.05.

### 2.3. The Third Experiment Was Carried out with Adults of Pelophylax ridibundus, of Both Sexes, Harvested from the Lakes Bordering the City of Piteşti

After capture, the animals were kept in glass water tanks with clean water that was changed daily to avoid the accumulation of toxins. The laboratory acclimation of the animals lasted five days, thus avoiding the sudden death of some specimens.

The animals were organized into two lots:

The control group, consisted of 10 individuals of *Pelophylax ridibundus*, males and females, untreated, and kept in tap water, which was changed daily. The control group was divided into two sublots: one made up of specimens kept at 6–8 °C and another made up of specimens kept at 18–20 °C. The specimens of both sublots were intraperitoneally injected with physiological saline for poikilotherms (6.5‰), with one injection every two days for three weeks.

The test group, consisted of 10 individuals of *Pelophylax ridibundus*, males and females, treated with Actara 25 WG by intraperitoneal injections, with one injection every two days in a three-week schedule. The animals were kept under conditions similar to those for the control group, and they were divided into two sublots: one consisting of treated specimens kept at a temperature of 6–8 °C and another made up of treated specimens kept at a temperature of 18–20 °C.

The control and test groups were composed of specimens that had approximately the same weight (40 g ± 5) and the same sex distribution. Throughout the experiment, the animals were fed ad libitum.

The injection volume for each individual was determined using the following formula:

(1) $$V=\frac{D\times W}{C}$$

where *V* = the injection volume (in mL), *D =* the required dose (in mg/kg), *W =* the body weight of each individual (in kg), and *C =* the concentration of the stock substance (in mg/mL). The required dose was 0.5 mg/kg.

At the end of the treatment, the animals were anesthetized with chloroform and were spinalized by a method indicated by Picoş and Năstăsescu [30]. Tissue samples were fixed in 8% neutral formalin for poikilotherms for 24 h and were processed using a graded ethanol series and embedded in paraffin. Paraffin section were cut 5 μm thick slices using a rotary microtome (Slee Maintz Cut 5062) and stained with hematoxylin (H) as a general screening method and Sirius red [32] for collagen stain (fibrosis). The sections were viewed and photographed using an Olympus microscope with an attached camera.

All the experiments were conducted in accordance with national and international guidelines of the European Parliament and the Council on the protection of animals used for scientific purposes according to Directive 2010/63/EU [33].

The study was conducted with the approval of the local Committee of Bioethics according to the Romanian law 205/2004 art.7, 18, 22 and regulation number 143/400/2002 for the care and use of animals for research purposes.

## 3. Results and Discussion

### 3.1. The Action of Actara 25 WG Insecticide on Oxygen Consumption in Carassius gibelio

Fish are sentinel organisms that respond to changes at various structural levels, from cellular, physiological, biochemical, genetic, and histological factors to stressors present in the environment [34].

Monitoring the oxygen consumption of aquatic organisms can be considered a better method for assessing the toxicity of a substance than performing acute toxicological tests (determining the survival of different species), as it results in low concentrations of the toxic substance [35].

Fish respond to environmental toxic changes by adapting their metabolic functions [36]; they have been successfully used as a model to study the negative effects of various pesticides on the environment [37].

The insecticide thiamethoxam affects the energy metabolism of fish due to its effects on both the respiratory system and the effects on cellular respiration [38].

Except for at the lowest tested concentration (0.064 mg/L), where there were insignificant increases in oxygen consumption within the first 24–48 h of exposure, the Actara 25 WG insecticide had the effect of reducing the energy metabolism of the Prussian carp (Table 1).

The strongest inhibitory effect of the insecticide was observed 24 h after the immersion of the Prussian carp in the toxic solution; the percentage decreases in this physiological index were 14.07%, 18.48%, and 26.52%, respectively, compared to the values recorded before the fish were immersed into the insecticide. After 72–96 h from the start of the experiments, the average values of oxygen consumption stabilized, and the changes occurring in the last seven days were insignificant for all the tested concentrations. In addition to the experimental variants performed at 6–8 °C, the Actara 25 WG insecticide at a concentration of 0.064 mg/L did not produce any significant effects on the Prussian carp. At the other three concentrations, the product had an inhibitory effect on oxygen consumption in the Prussian carp.

The increased metabolism of the fish at higher temperatures might lead to a higher uptake and higher toxicity of the substances at higher temperatures; the higher metabolism could also lead to a higher (de)toxification of the substances, which might be expected to be predominantly true for the pesticides, or the increased temperature in the water could lead to faster degradation of the pesticides outside the fish, producing more toxic or less toxic metabolites [39].

Similar to the evolution of energy metabolism in the Prussian carps intoxicated with Actara 25 WG at 18–20 °C, and in the cold variants, the strongest effect occurred 24 h after initiating the experiments, with the percentage reduction of this index being less pronounced than that at higher temperature (the average values recorded were 9.69%, 14.73%, and 19.26%, respectively, compared to the control values). The reductions in oxygen consumption in the cold-mounted variants were significant at the *p* < 0.05 level, except for those determined at the concentration of 0.064 mg/L.

The Prussian carp intoxicated with Actara 25 WG (at concentrations of 0.128, 0.256, and 0.512 mg/L) showed, after 14 days, a decrease in energy metabolism of 39.23–46.44% (significant at the *p* < 0.05 level) at room temperature and of 19.92–40.37% at low temperature.

The decrease in oxygen consumption can also be attributed to the degradation of the gills under the action of the insecticide. At a concentration of 20 mg of thiamethoxam/L, Georgieva found histological alterations in *Cyprinus carpio* gills: lamellar lifting, edema, and the proliferation of filamentous epithelial cells [40].

### 3.2. The Action of Actara 25 WG Insecticide on the Respiratory Rate in Carassius gibelio

The first concentration of the tested insecticide (0.064 mg/L) had no significant effect on the respiratory rate of the Prussian carps maintained at room temperature throughout the experiment. For the other three tested concentrations, the insecticide increased the respiratory rate within the first 24–48 h (Table 2) from the introduction of the fish into the solutions (by 19.23%, 16.71%, and 4.79%, respectively, compared to the control values).

Increases in the frequency of respiratory movements represent the fish’s attempt to compensate for the decrease in the amount of oxygen taken up by the gills (whose permeability is affected by the insecticide) under the conditions of diminishing oxygen consumption.

The subsequent evolution of this parameter is similar to that recorded for the energy metabolism, namely, a continuous reduction, with the average values determined at the end of the experiments being lower than those recorded before the introduction of the fish into the toxic environment (there were no major differences between the three groups: 88.57%, 83.66%, and 82.17%, respectively, compared to the control values).

The much weaker effect of the Actara 25 WG insecticide at low temperatures was also demonstrated by the evolution of the respiratory rate in the Prussian carps maintained at 6–8 °C and exposed to the action of the insecticide at different concentrations.

There were no significant changes in this physiological index during the entire exposure of the Prussian carps to Actara 25 WG at a concentration of 0.064 mg/L. For the other three tested concentrations, after a slight increase (relevant for the significance threshold of *p* < 0.05) in the first 24–72 h (by 12.1%, 12.67%, and 21.42%, respectively, compared to the control values), the respiratory rhythm returned to normal.

Any injury to the gills is followed by a chain of destructive events, which ultimately leads to the initialization of “respiratory shock”.

Stoyanova et al. [41] found pronounced alterations in the gill histological structure of bighead carp (*Hypophthalmichthys nobilis)* exposed to thiamethoxam in a short-term laboratory condition (96 h) at 6 mg/L, 10 mg/L, and 20 mg/L concentrations: lamellar lifting, the proliferative changes concerning the squamous epithelium and the glandular cells, and edema at the base of the secondary lamellae. The authors also reported changes in the circulatory system at 20 mg/L thiamethoxam concentration: vasodilatation along the length of the blood vessel in the secondary lamellae and vasodilatation of the central venous sinus.

The histological changes in the gill structure could be a reaction of the fish to toxicant intake or an adaptive response in order to prevent the entry of the pollutants through the gill surface [40].

After two weeks of exposure to the insecticide Actara 25 WG at a concentration of 0.064 mg/L, the number of erythrocytes decreased significantly (at room temperature by 14.54%; at low temperatures by 16.2%). The decrease in erythrocyte counts as a result of exposure to thiamethoxam at a concentration of 12.5 mg/L water, reported by Barnali and Nath at *Oreochromis niloticus* (Trewavas) after 7 and 14 days of exposure, respectively, with a more pronounced effect after 7 days of exposure to insecticide [42].

Thiamethoxam exposure even at sub-lethal levels (50, 100, 200, and 400 mg/kg B.W.) have toxic effects on various blood parameters in broiler chicks [43]; hematological parameters including total erythrocytes, hemoglobin, and hematocrit values were significantly decreased with the increase in dose rate of thiamethoxam. This might be due the effect of pesticides on hemopoietic system [44]. Thiamethoxam administration (87.73 mg/kg b.w. for 28 days) on male Swiss albino mice produced significant reductions in erythrocyte count and hemoglobin concentration [45].

### 3.3. Influence of Fish Size on the Toxicity of Actara 25 WG Insecticide in Prussian Carp

**Figure 1:**
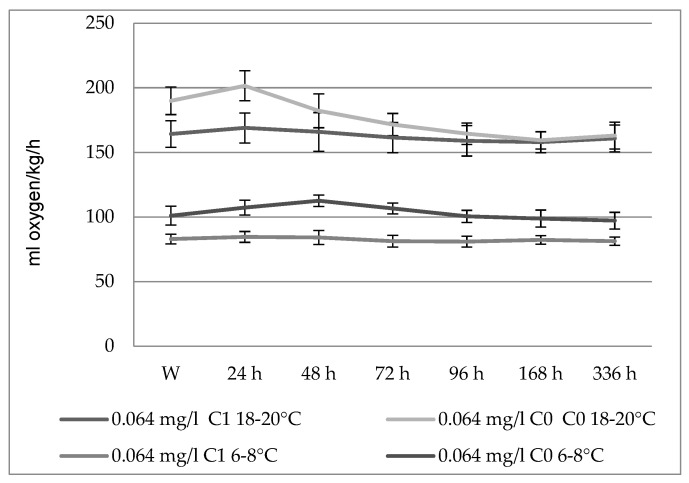
Influence of fish size on the oxygen consumption in Prussian carp exposed to Actara 25 WG insecticide and the standard deviation.

There were no differences in the evolution of the oxygen curves in Prussian carps of different sizes exposed to the action of the insecticide at a concentration of 0.064 mg/L (Figure 1). Virtually no significant changes in energy metabolism occurred at this concentration until 72 h after the introduction of the fish into the toxic environment.

**Figure 2:**
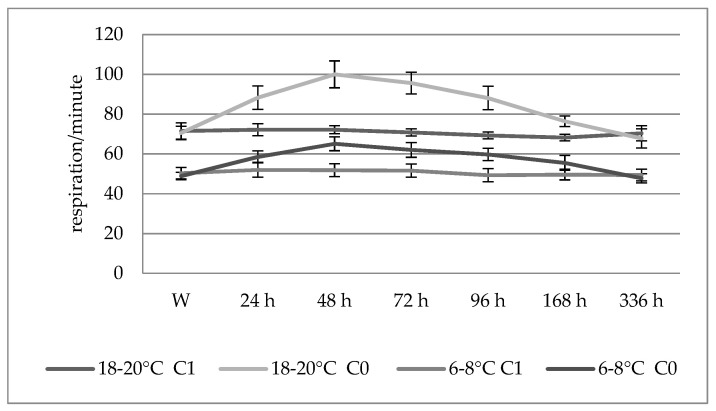
Influence of fish size on the average respiratory rate in Prussian carp exposed to Actara 25 WG insecticide and the standard deviation.

Within the experimental variants conducted on the Prussian carps, there were differences regarding the evolution of the frequency of respiratory movements between fish of different sizes exposed to Actara 25 WG at a concentration of 0.064 mg/L (Figure 2).

While the larger Prussian carps did not show significant changes in the respiratory rate, the smaller ones showed a higher respiratory rate 24–48 h after exposure, with significance at the *p* < 0.05 level (105.17% and 111.28%, respectively, compared to the control values); after passing this interval, the respiratory rate returned to the normal values, with the values at the end of the experiment being insignificantly different from the control values.

The higher resistance of the fish to the action of the insecticide at low temperatures can be explained by a lower permeability of the branchial tissue for the toxic substance (the reduction in oxygen consumption also contributed to the penetration of the toxin into the fish’s body in smaller amounts); it is possible that there are fewer branchial lesions at this temperature, probably due to the lower solubility of the product.

Except for the lowest concentration tested (0.064 mg/L), at the end of the acute test (96 h) mortality was recorded in all variants mounted at room temperature.

At the end of the experiment, mortality in lots was lower at low temperatures (40% mortality at highest concentration–0.512 mg/L) and higher at room temperature (70% mortality at the highest concentration–0.512 mg/L).

### 3.4. The Action of Actara 25 WG Insecticide on the Lung in Pelophylax ridibundus

The intraperitoneal administration of the Actara 25 WG insecticide at a concentration of 0.5 mg/g body weight and 0.125 mg of thiamethoxam/g body weight, respectively, caused histological changes in the lung.

**Figure 3:**
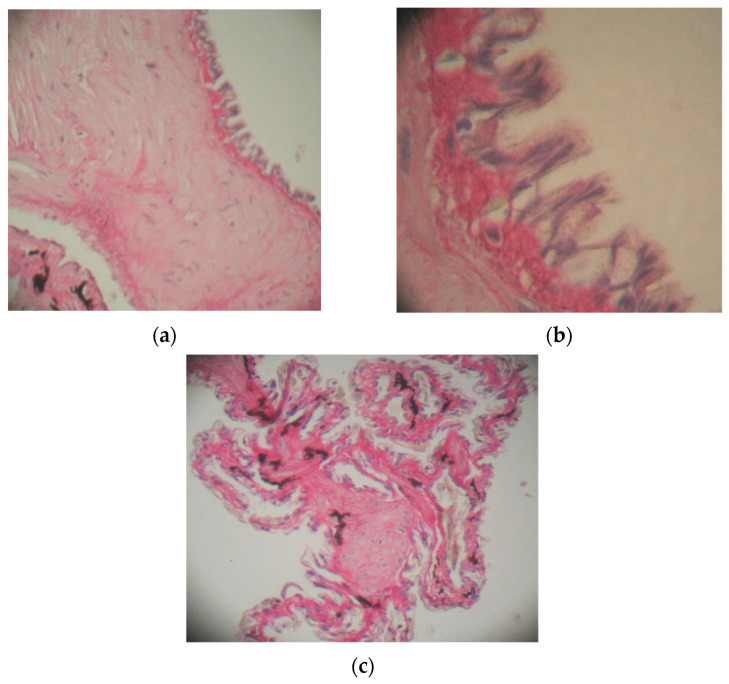
Lung of *Pelophylax ridibundus* in specimens treated with Actara 25 WG insecticide and kept at 6–8 °C: (**a**) thickening of the first-order septa bordered by a pseudostratified epithelium, scale bar 25 μ 100×; (**b**) cylindrical cells with cilia, with basal cells and caliciform cells, scale bar 50 μ 400×; (**c**) conjunctive septa of the second and third order, scale bar 25 μ 100×. H-Sirius red.

In the case of animals kept at a temperature of 6–8 °C, there was a marked thickening of the conjunctive longitudinal septa of the first order compared with the control specimens (Figure 3a).

Numerous smooth muscle fibers and elastic fibers are found in the walls of the conjunctival septum. The first-order septum is bordered by a pseudostratified epithelium composed of ciliated cylindrical cells that have an elongated nucleus, oriented towards the apical region, and caliciform cells that have numerous granulations in the cytoplasm and basal cells (Figure 3b). The conjunctival septum of the third order is surrounded on both sides by a respiratory epithelium composed of pneumocytes and caliciform cells, next to which the blood capillaries are located (Figure 3c).

Under the toxic action of thiamethoxam, there was an increase in the number of caliciform cells; these cells react by increasing the volume and accumulation in the cytoplasm of a granular material, a change that causes their activity to cease.

**Figure 4:**
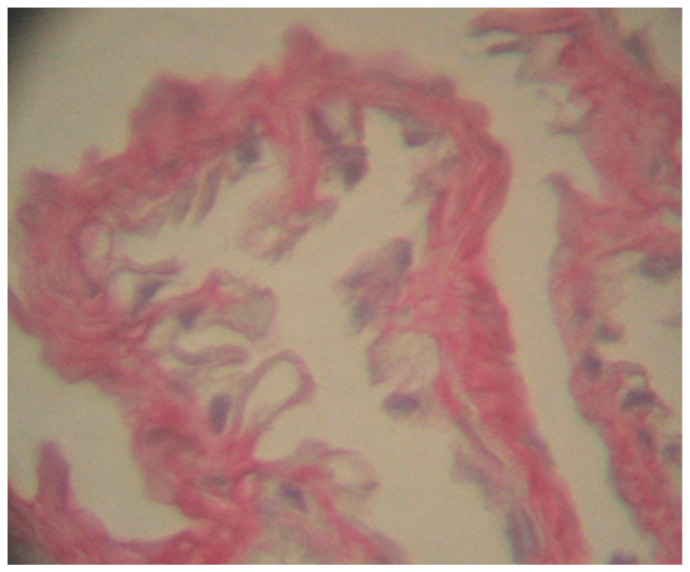
Respiratory features in *Pelophylax ridibundus* lung hypertrophied caliciform cells with secretory granules in the cytoplasm and non-functional pneumocytes, scale bar 50 μ. 400×; Perls, H-Sirius red.

The pneumocytes also show reduced activity, being surrounded by lysed erythrocytes that were not capable of performing respiratory gas exchange (Figure 4).

**Figure 5:**
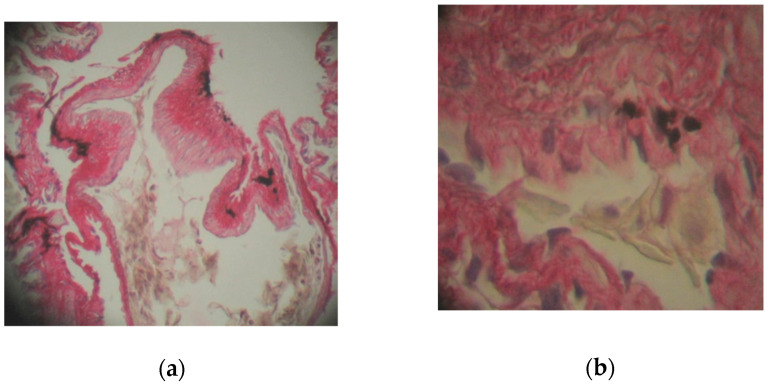

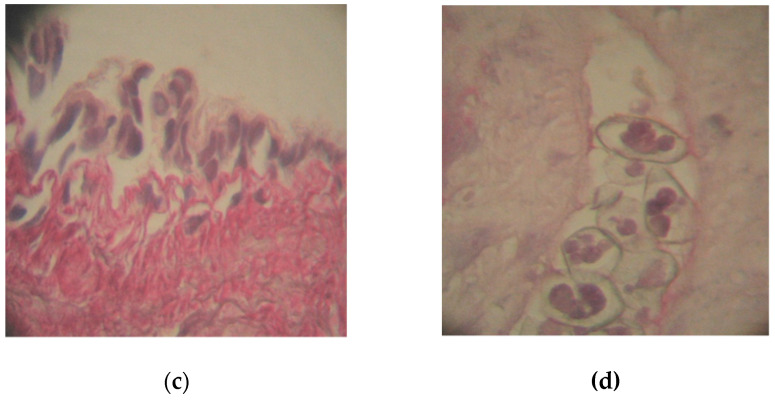
Cross-sections of the lung of *Pelophylax ridibundus* in specimens treated with Actara 25 WG insecticide and kept at 18–20 °C: (**a**) bleeding areas in the pulmonary parenchyma (H), scale bar 25 μ 100×; (**b**) blood capillaries with lysed erythrocytes; (**c**) the tendency of the epithelium to exfoliate; (**d**) the presence of leukocyte infiltrates, scale bar 50 μ 400×. H-Sirius red.

At 18–20 °C, the histological changes were more pronounced, probably due to the higher metabolism of the frogs. Hemorrhagic areas appeared in the pulmonary parenchyma (Figure 5a); the conjunctive septa of the first and second order were very thick.

In the capillary, the erythrocytes were lysed, and the presence of infiltrated leukocytes was observed as a result of the inflammatory process initiated by the toxin (Figure 5b,d). Other blood capillaries were disorganized. Additionally, the epithelium lining the conjunctival septa tended to exfoliate (Figure 5c).

The existence of a large number of caliciform cells is explained by the need for the respiratory-type epithelium to have a larger quantity of mucus, with the function of protecting the deep structures.

All of these changes were associated with the presence of thiamethoxam in the respiratory cells, which caused the exchange of respiratory gases to be blocked.

The ratio between the diameter of the nuclei and the diameter of the pneumocytes (Table 3) was found to be higher in animals treated with Actara 25 WG and maintained at 18–20 °C.

Increases in the value of this ratio are a consequence of increases in the diameter of the nucleus compared to the diameter of the cell under exposure to sublethal concentrations of the investigated insecticide.

From the analysis presented, it can be concluded that the toxic effect is more pronounced at 18–20 °C than at 6–8 °C; this is due to the fact that amphibians are poikilothermic organisms that reduce their activity at low temperatures.

Al-Sharqi [46] observed histopathological changes in the livers and kidneys of mice induced by the toxic action of Actara 25 WG, such as disturbances in the hepatic lobule structure, hepatocyte hypertrophy with severe inflammatory cell infiltration, Kupffer cell proliferation, coagulative necrosis, and hydropic degeneration. Histopathological lesions of the kidney showed lobulated glomeruli, a large-area hemorrhage, congested blood vessels that showed thickening in their walls, degenerative changes, and the infiltration of inflammatory cells.

The metabolism of xenobiotic substances can be considered a detoxification process, by means of which the fat-soluble substances are transformed by oxidation and conjugation, and then eliminated by excretion. There are xenobiotic substances that, as a result of their metabolism, become much more toxic than the parental molecules. An example is thiamethoxam, whose metabolites are much more toxic, especially hepatotoxic, than the substance itself, showing tumorigenic effects [47]. Numerous studies also show that neonicotinoid insecticides induce oxidative stress by unbalancing the antioxidant balance [17,48,49,50,51].

## 4. Conclusions

The Actara 25 WG insecticide reduces oxygen consumption in Prussian carp, with a maximum effect at 24–48 h after exposure for all the variants of concentrations (except the lowest concentration) and the metabolic decrease is more pronounced for fish kept at room temperatures (18–20 °C).

The respiratory rate of the intoxicated fish increased in the first phase, with a variable duration of the experimental conditions, after which the values of this physiological index fell below the reference values. The smaller Prussian carps (C_0_) were more sensitive than the larger carps (C_1_) to the action of the Actara 25 WG insecticide.

Although there are no significant differences in oxygen consumption depending on the age of the fish, regardless of the thermal level tested, the smaller fish were more sensitive, showing significant increases in respiratory rate in the first 24–46 h of insecticide exposure. However, at the end of the exposure (after 336 h), the fish kept at low temperature adapted better, and the values of this physiological index returned to their normal limits. These changes occurred when the fish were acclimatized to the temperature, so we wonder what will happen in the event of a sudden change in water temperature, a situation common in nature due to climate change and which could irreversibly influence energy metabolism, with serious effects on the ecosystem.

And in the case of the frog experiment, our results show that significant changes occurred in animals exposed to room temperature. The histopathology of the lung illustrates the thickening of the conjunctival septum, an increase in the number of mucous cells, and an increase in the ratio between the diameter of the nucleus and the diameter of the pneumocyte. Frogs treated with insecticide and kept warm showed greater histological changes than those exposed to low temperatures. We have encountered the same effects as in fish, which strengthens our belief that, in the event of water contamination with insecticides, the sudden rise in temperature can have significant effects on the common organisms and on the functioning of the ecosystem.

## Figures and Tables

**Table 1:** Variations in the average oxygen consumption (mL oxygen/kilogram/hour) and the standard deviation of the Prussian carp exposed to Actara 25 WG insecticide at different concentrations.

T (°C)	Actara 25 WG Concentration (mg/L)	Before Exposure	24 h	48 h	72 h	96 h	168 h	336 h
18–20	0.064	164.3 ± 10.65	169 ± 11.58	165.9 ± 13.05	161.5 ± 8.64	159 ± 8.28	158 ± 6.64 *	160.9 ± 10.38 *
	0.128	150 ± 9.42	128.9 ± 7.76 *	115.9 ± 4.48 *	110.88 ± 3.14 *	102.77 ± 5.95 *	90.88 ± 4.8 *	92.66 ± 7.56 *
	0.256	160.8 ± 3.45	131.1 ± 5.56 *	113.44 ± 4.95 *	104.77 ± 4.91 *	94.87 ± 7.39 *	88 ± 4.16 *	86.14 ± 9.8 *
	0.512	157.7 ± 5.25	115.88 ± 3.78	103.42 ± 5.22	94.4 ± 5.5	90.25 ± 10.21 *	90.5 ± 16.52 *	89.33 ± 4.16 *
	0 (control lot)	165 ± 9.54	167 ± 2.42	166 ± 2.55	164 ± 3.65	166 ± 1.56	168 ± 4.82	166 ± 3.46
6–8	0.064	82.9 ± 3.78	84.6 ± 4.19	84.1 ± 5.42	81.3 ± 4.52	80.9 ± 4.17	82.3 ± 3.33	81.3 ± 3.26
	0.128	81.4 ± 4.03	73.6 ± 3.47 *	70.7 ± 3.94 *	69.9 ± 2.99 *	68.3 ± 3.94 *	67.3 ± 4.47 *	66.2 ± 3.6 *
	0.256	84.9 ± 2.92	72.4 ± 2.67 *	67.4 ± 2.79 *	64.88 ± 3.51 *	62.33 ± 4.18 *	60.62 ± 4.27 *	61.25 ± 3.25 *
	0.512	88.3 ± 3.19	71.3 ± 2.75 *	64.22 ± 3.52 *	58.37 ± 4.8 *	52.28 ± 2.98 *	53.28 ± 2.92 *	52.66 ± 1.63 *
	0 (control lot)	84 ± 2.54	86 ± 3.25	84 ± 4.82	86 ± 3.64	85 ± 3.25	82 ± 2.45	83 ± 2.55

* The mean difference is significant at the 0.05 level.

**Table 2:** Variations in the average respiratory rate (breaths/minute) and the standard deviation of the Prussian carps exposed to Actara 25 WG insecticide at different concentrations.

T (°C)	Actara 25 WG Concentration (mg/L)	Before Exposure	24 h	48 h	72 h	96 h	168 h	336 h
18–20	0.064	71.5 ± 4.08	72.2 ± 3.04	72.2 ± 2.04	70.8 ± 1.75	69.3 ± 1.76	68.2 ± 1.68	70.4 ± 3.73
	0.128	67.6 ± 3.65	79.3 ± 5.29	80.6 ± 5.44 *	72.44 ± 5.91	64.55 ± 3.97 *	61.11 ± 4.22 *	59.88 ± 4.67 *
	0.256	71.2 ± 1.75	83.1 ± 4.01 *	82.11 ± 4.13 *	73.55 ± 6.36	68.25 ± 5.7 *	52.828 ± 2.82 *	59.57 ± 2.99 *
	0.512	72.2 ± 4.93	75.66 ± 1.22	70.85 ± 2.73	65.6 ± 2.19	62 ± 2.82 *	58 ± 4.24 *	59.33 ± 5.77 *
	0 (control lot)	72 ± 2.45	73 ± 1.25	73 ± 064	75 ± 2.52	71 ± 3.55	73 ± 2.45	73 ± 3.45
6–8	0.064	50.3 ± 2.86	52 ± 3.74	51.8 ± 3.25	51.6 ± 3.33	49.3 ± 3.26	49.6 ± 2.67	49.4 ± 2.95
	0.128	47.9 ± 1.91	53.7 ± 1.82	52.9 ± 1.72 *	50.3 ± 2.05	47.3 ± 2.49	44.7 ± 1.63 *	43.11 ± 2.97 *
	0.256	48.9 ± 2.23	55.1 ± 1.44	54.4 ± 1.5 *	50.88 ± 2.36	47.66 ± 2.29	46 ± 2.87	44.12 ± 2.53
	0.512	47.6 ± 2.45	57.8 ± 4.58 *	54.55 ± 3.24 *	45.37 ± 2.26	42.28 ± 2.42 *	39.85 ± 4.09 *	41.66 ± 1.86
	0 (control lot)	51 ± 2.45	52 ± 1.25	50 ± 3.25	51 ± 2.65	50 ± 4.15	51 ± 2.67	50 ± 3.25

* The mean difference is significant at the 0.05 level.

**Table 3:** The ratio between the diameter of the nuclei and the diameter of the pneumocytes.

Batch	Number of Measurements	Average and Standard Deviation
C 6–8 °C	200	0.58 ± 0.029
Lot I	200	0.67 ± 0.019
C 18–20 °C	200	0.75 ± 0.022
Lot II	200	0.88 ± 0.018

## Data Availability

Not applicable.
